# Antioxidant Effects of *Satureja hortensis* L. Attenuate the Anxiogenic Effect of Cisplatin in Rats

**DOI:** 10.1155/2019/8307196

**Published:** 2019-07-29

**Authors:** Igor Kumburovic, Dragica Selakovic, Tatjana Juric, Nemanja Jovicic, Vladimir Mihailovic, Jelena Katanic Stankovic, Nikola Sreckovic, Davor Kumburovic, Vladimir Jakovljevic, Gvozden Rosic

**Affiliations:** ^1^Department of Physiology, Faculty of Medical Sciences, University of Kragujevac, Kragujevac, Serbia; ^2^Faculty of Agriculture, University of Novi Sad, Novi Sad, Serbia; ^3^Department of Histology and Embryology, Faculty of Medical Sciences, University of Kragujevac, Kragujevac, Serbia; ^4^Department of Chemistry, Faculty of Science, University of Kragujevac, Kragujevac, Serbia; ^5^Department of Human Pathology, 1st Moscow State Medical University IM Sechenov, Moscow, Russia

## Abstract

Numerous adverse effects of cisplatin-based therapy are usually accompanied by enhanced oxidative damage and cell apoptosis in various tissues. Even neurotoxic manifestations of cisplatin administration, such as the anxiogenic effect, appear along with the increased oxidative stress and apoptotic indicators in certain brain regions. Thirty-five Wistar albino male rats were divided into seven groups: control, cisplatin (received a single dose of cisplatin: 7.5 mg/kg), three groups with oral administration of *Satureja hortensis* L. methanolic extract (SH) (low: 50 mg/kg, middle: 100 mg/kg, and high dose: 200 mg/kg) along with cisplatin application, a group with the extract in high dose alone, and a silymarin group (cisplatin and silymarin: 100 mg/kg), in order to evaluate the antioxidant effects of SH on cisplatin-induced increase in the anxiety level. After completing 10-day pretreatments, behavioral testing was performed in the open field and the elevated plus maze, followed by an investigation of oxidative stress and apoptosis parameters in hippocampal tissue samples. Cisplatin administration resulted in anxiogenic-like behavior, increased lipid peroxidation, and proapoptotic markers accompanied by the decline in antioxidant and antiapoptotic defense. The administration of extract alone did not significantly alter any of the estimated parameters. When applied along with cisplatin, SH in a dose of 100 mg/kg induced the significant anxiolytic effect with concomitant recovery of antioxidant and antiapoptotic activity indicators, while both lower and higher doses of the extract failed to improve the adverse effects of cisplatin administration. The beneficial effects of the middle dose of SH were equivalent to the same dose of silymarin, as a “golden standard.” Our results indicate that the antioxidant supplementation with SH in an optimal dose significantly improved the oxidative status and it had antiapoptotic effect in the rat hippocampus disturbed by cisplatin administration, which was accompanied with attenuation of cisplatin-induced anxiogenic effect.

## 1. Introduction

Generation of reactive oxygen species (ROS) in brain tissue is considered to be the main process linked to the appearance of a broad spectrum of psychiatric disorders [1]. A chemotherapeutic drug, cisplatin, although considered to be one of the most promising chemotherapeutics in the treatment of various malignancies, exerts morphological or behavioral impairments in brain tissue [2]. The primary evidence of neurotoxicity of cisplatin is decreased activity of antioxidant enzymes with concomitant depletion in the glutathione level, elevated lipid membrane peroxidation, and mitochondrial dysfunction. This indicates that cisplatin causes overproduction of ROS and imbalance between oxidant-antioxidant levels, consequently leading to the insufficient level of antioxidants to counteract the raised ROS in tissue [3]. Besides, cisplatin-induced neurotoxicity has been shown to result in numerous clinical forms. Although the majority of the investigated mechanisms focused on the manifestations of peripheral nerve injury [4], recent investigations have paid more attention to the effects on the central nervous system. Therefore, it has been reported that various protocols of cisplatin administration may affect mood regulation [5], as well as cognitive and perceptual impairments [6]. Furthermore, it has been proposed that the described behavioral alterations are connected with an underlying increase of oxidative stress [7] in specific brain regions involved in the control of those features. Also, Manohar and coworkers demonstrated that cisplatin promotes cell death in the hippocampus by increasing the expression of proapoptotic genes while reducing the expression of antiapoptotic genes [8].

Phytochemicals and plant-based products have been widely applied in treatments of various types of neurological disorders, due to their safety and the absence of side effects [9]. *Satureja hortensis* L. (summer savory, garden savory) is an annual shrub belonging to the genus *Satureja* of the Lamiaceae family. Summer savory is native to North America as well as warmer regions, such as the Mediterranean area and Southern Europe [10]. Due to its flavoring properties, the aerial parts of some *Satureja* species are mostly used as a culinary spice [11], but their medical benefits on human health are also remarkable. In traditional medicine, *S. hortensis* has been widely applied to relieve muscle pain, as a carminative and as a medicament in the treatment of diarrhea and digestion impairments [12]. Modern phytochemical analysis revealed a wide range of bioactive constituents in *S. hortensis*: monoterpenoids, carvacrol, thymol, and *p*-cymene as the main compounds in volatile oil [13] and phenolics, especially a high amount of rosmarinic acid, as major active principles in the extract [14]. Considering the different active compounds that are present in *S. hortensis*, the exceptional potential of essential oil and extracts of summer savory has been reported in *in vitro* and *in vivo* studies. Recent studies showed that *S. hortensis* exhibited notable antioxidant, antimicrobial, antiparasitic, anti-inflammatory, antinociceptive, antiviral, anti-Alzheimer (acetyl- and butyrylcholinesterase inhibitory activity), and hepatoprotective properties [15, 16]. Our earlier study showed the amelioration effect of *S. hortensis* methanolic extract against cisplatin-induced hepatorenal and testicular toxicity [17]. Similarly, it was demonstrated that administration of *S. hortensis* extract could prevent alterations in cisplatin-induced oxidative damage in several tissues [18]. There are no studies on toxicity of *S. hortensis* extract, but Fierascu et al. [15] recently reported that *S. hortensis* tea or infusion can be safely consumed, but essential oil of summer savory may cause mild dermal irritations. Previous investigations showed that rosmarinic acid, the main phenolic compound in *S. hortensis* extract, produced antidepressive and anxiolytic effects [19], and therefore, the following investigations were performed in order to evaluate the effects of *S. hortensis* methanolic extract (SH) on the cisplatin-induced increase in the anxiety level and oxidative stress in hippocampal tissue.

## 2. Materials and Methods

### 2.1. Extract Preparation

Aerial parts of *Satureja hortensis* L. were collected during the full flowering period in August 2015 in village Borač (municipality of Kragujevac, Republic of Serbia). A voucher specimen (No. 121/15) was deposited in the Herbarium of the Department of Biology and Ecology, Faculty of Science, University of Kragujevac, Kragujevac, Serbia, after the identification of species. Plant material was air-dried at room temperature. The methanolic extract of *S. hortensis* was prepared according to the previously published procedure [17]. Phytochemical characterization of methanolic extract using HPLC/DAD/(-)HESI-MS/MS analysis was previously published in the study by Boroja and coworkers, and this methanolic extract was investigated in the presented paper, as a part of the same study [17].

### 2.2. Animals and Treatment

Thirty-five male Wistar albino rats (two months old, 200-250 g, obtained from the Military Medical Academy, Belgrade, Serbia) were included in this study. The animals were housed in groups of five per cage in suitable environmental conditions (23 ± 1°C and 12/12 h light/dark cycles) and had free access to food and tap water. Animals were divided into seven equal groups and underwent pretreatment protocols, as follows: the control group received water for 10 days and a single intraperitoneal (i.p.) application of 0.5 mL normal saline on the 5th day; the CIS group received water for 10 days and a single dose of cisplatin (7.5 mg/kg b.w., i.p.) on the 5th day; the CIS+E50 group received *S. hortensis* extract (50 mg/kg b.w.) orally for 10 days and a single dose of cisplatin (7.5 mg/kg b.w., i.p.) on the 5th day; the CIS+E100 group received *S. hortensis* extract (100 mg/kg b.w.) orally for 10 days and a single dose of cisplatin (7.5 mg/kg b.w., i.p.) on the 5th day; the CIS+E200 group received *S. hortensis* extract (200 mg/kg b.w.) orally for 10 days and a single dose of cisplatin (7.5 mg/kg b.w., i.p.) on the 5th day; the CIS+silymarin group received silymarin (100 mg/kg b.w.) orally for 10 days and a single dose of cisplatin (7.5 mg/kg b.w., i.p.) on the 5th day; and the E200 group received *S. hortensis* extract (200 mg/kg b.w.) orally for 10 days.

The final concentration of extract doses was calculated on the basis of average water intake in the previous 24 hours.

### 2.3. Behavioral Testing

Twenty-four hours after completing the protocols, the animals were allowed to acclimate in a testing room (approximately at 8 am) for at least 1 h, and behavioral testing was performed in an open field (OF) and the elevated plus maze (EPM), as previously described [20]. The following parameters were estimated in the OF test: cumulative duration in the center zone (CDCZ, in seconds), frequency in the center zone (FCZ), total distance moved (TDM, in cm), the percentage of time moving (%TM), and the number of rearings during 5 minutes of testing. Immediately after completing the OF test, the rats were placed in the EPM in order to obtain different parameters: cumulative duration in open arms (CDOA, in seconds), frequency to open arms (FOA), TDM (in cm), %TM, the number of rearings, the number of head-dippings (HD), and the number of total exploratory activity (TEA) episodes. Both tests were recorded, and video files were analyzed using EthoVision software (XT 12, Noldus Information Technology, The Netherlands).

After completing behavioral testing, the rats were decapitated following a short-term narcosis induced by intraperitoneal application of ketamine (10 mg/kg) and xylazine (5 mg/kg), the brains were quickly removed, and hippocampal tissue was isolated for further analysis.

### 2.4. The Estimation of Oxidative Stress Parameters in the Hippocampus

The hippocampus tissue homogenates were prepared in phosphate-buffered saline (PBS, pH 7.4) and centrifuged at 4000 rpm for 15 min at 4°C. The obtained supernatants were used for the determination of catalase (CAT) and superoxide dismutase (SOD) activities, as well as reduced glutathione (GSH) and thiobarbituric acid reactive substance (TBARS) levels. The activity of CAT in tissue homogenates was determined by monitoring the decomposition rate of hydrogen peroxide according to the method described by Beers and Sizer [21]. The measurement of SOD activity in hippocampus tissue homogenates was based on the colorimetric reaction inhibition of adrenochrome formation from adrenalin according to the method of Misra and Fridovich [22]. CAT and SOD activities in tissue homogenates were expressed as unit per milligram of protein (U per mg of proteins). The reduced glutathione (GSH) level in homogenates was estimated by Ellman's procedure and expressed as mg GSH per gram of proteins [23]. The thiobarbituric acid reactive substance (TBARS) level in samples was determined by the method of Ohkawa and coworkers and expressed as nmol MDA per mg of proteins [24]. The Lowry et al.'s method [25] was used for the determination of protein concentrations in tissue homogenates, using bovine serum albumin (BSA) as a standard. All spectrophotometric measurements were performed using a UV-Vis double beam spectrophotometer (model Halo DB-20S, with a temperature controller, Dynamica GmbH, Dietikon, Switzerland).

### 2.5. RNA Isolation and Real-Time PCR Analysis

Total RNA was extracted from hippocampal tissue using the TRIzol reagent (Invitrogen, CA) according to the manufacturer's instructions. For reverse transcription, a High-Capacity cDNA Reverse Transcription Kit (Applied Biosystems, CA) was used. Quantitative RT-PCR was performed using the Thermo Scientific Luminaris Color HiGreen qPCR Master Mix (Applied Biosystems, CA). Also, mRNA-specific primers for Bax, Bcl-2, caspase-3, and *β*-actin as a housekeeping gene (Invitrogen, CA) were used (Supplement 1). Quantitative RT-PCR reactions were done in the Mastercycler ep realplex (Eppendorf, Germany), and after data analysis, relative gene expression was calculated according to Livak and Schmittgen [26].

All research procedures were carried out in accordance with the European Directive for the welfare of laboratory animals No. 86/609/EEC and the principles of Good Laboratory Practice (GLP) and in accordance with the ARRIVE guidelines. All experiments were approved by the Ethical Committee of the Faculty of Medical Sciences, University of Kragujevac, Serbia.

### 2.6. Statistical Analysis

The data were presented as means ± S.E.M. After completing the tests for homogeneity (Levene's) and normality (Shapiro-Wilk), comparisons between groups were performed using one-way ANOVA, followed by Bonferroni post hoc analysis. Simple linear regression analyses were performed to analyze relationships between parameters obtained in behavioral tests and other analyses (oxidative stress and apoptosis indicators). Significance was determined at *p* < 0.05 for all tests. Statistical analysis was performed with SPSS version 20.0 statistical package (IBM SPSS Statistics 20).

## 3. Results

### 3.1. Antioxidant Supplementation Attenuates Cisplatin-Induced Anxiogenic Effect

**Figure 1:**
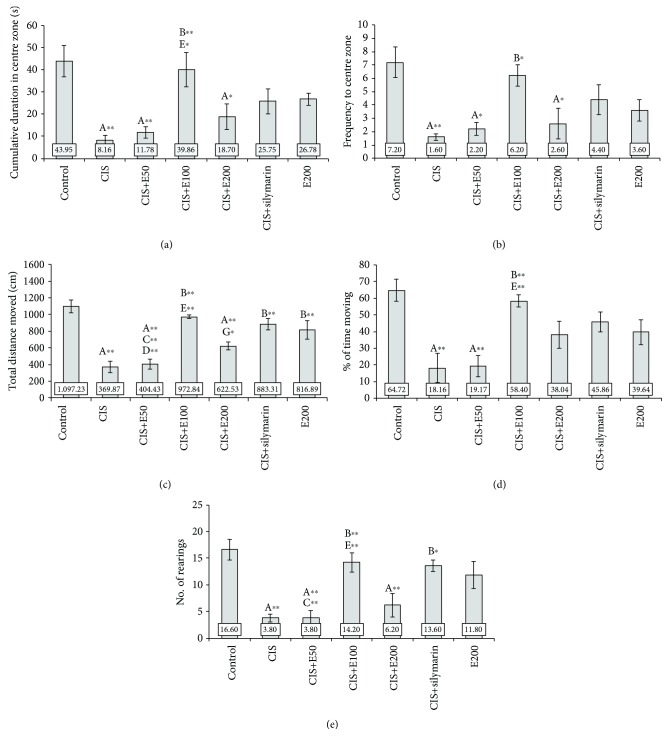
Parameters obtained in the open field (OF) test: (a) the cumulative duration in center zone (CDCZ), (b) the frequency to center zone (FCZ), (c) the total distance moved (TDM), (d) the percentage of time moving (%TM), and (e) the number of rearings. Values are the mean ± standard error of the mean (SEM); *n* = 5 per group. ∗ denotes a significant difference of *p* < 0.05; ∗∗ denotes a significant difference of *p* < 0.01. A: control vs. other groups; B: CIS vs. other groups; C: CIS+silymarin vs. other groups; D: E200 vs. other groups; E: CIS+E50 vs. CIS+E100; F: CIS+E50 vs. CIS+E200; G: CIS+E100 vs. CIS+E200.

**Figure 2:**
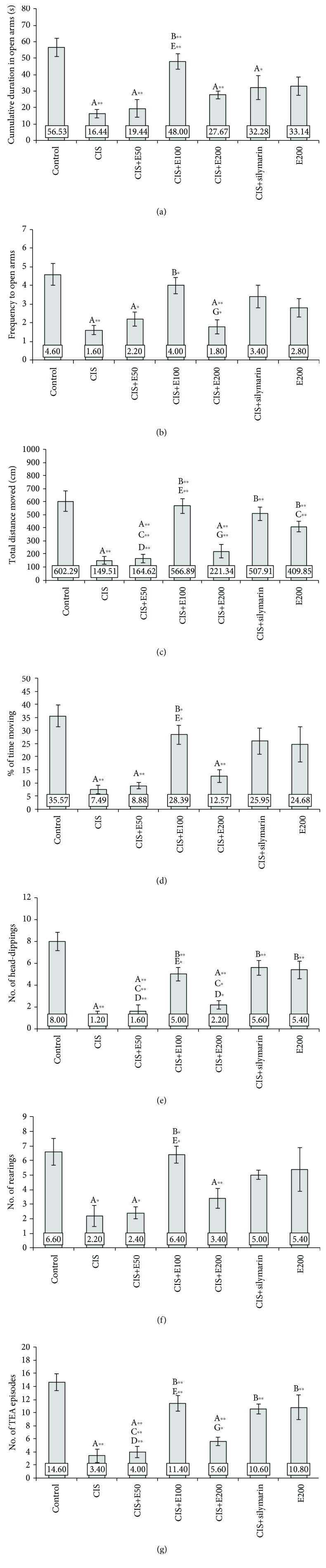
Parameters obtained in the elevated plus maze (EPM) test: (a) the cumulative duration in open arms (CDOA), (b) the frequency to open arms (FOA), (c) the total distance moved (TDM), (d) the percentage of time moving (%TM), (e) the number of head-dippings (HD), (f) the number of rearings, and (g) the number of TEA episodes. Values are the mean ± standard error of the mean (SEM); *n* = 5 per group. ∗ denotes a significant difference of *p* < 0.05; ∗∗ denotes a significant difference of *p* < 0.01. A: control vs. other groups; B: CIS vs. other groups; C: CIS+silymarin vs. other groups; D: E200 vs. other groups; E: CIS+E50 vs. CIS+E100; F: CIS+E50 vs. CIS+E200; G: CIS+E100 vs. CIS+E200.

Behavioral testing performed in this study revealed the anxiogenic-like effect of cisplatin in both OF (Figure 1) and EPM (Figure 2) tests. Cisplatin administration significantly decreased the principal indicators of the anxiety level, CDCZ (Figure 1(a)), and FCZ (Figure 1(b)), in the OF test (*F* = 6.527 and 5.530, respectively; df = 6, *p* < 0.01). The similar response to cisplatin, expressed as a significant decline in key parameters for anxiety level estimation in EPM, CDOA, and FOA (Figures 2(a) and 2(b); *F* = 8.204 and 6.027, respectively; df = 6, *p* < 0.01) was observed. Also, the overall locomotor activity in OF was significantly reduced following cisplatin application by means of TDM (Figure 1(c)) and %TM (Figure 1(d)) (*F* = 16.064 and 6.609, respectively; df = 6, *p* < 0.01), as it was in the EPM test, by means of the same parameters (Figures 2(c) and 2(d); *F* = 14.693 and 7.441, respectively; *p* < 0.01). At the same time, the exploratory activity was significantly reduced in OF (Figure 1(e); *F* = 8.980, *p* < 0.01) and EPM (Figures 2(e)–2(g)) that was manifested as decline in the number of HD (*F* = 15.590, *p* < 0.01), rearings (*F* = 4.920, *p* < 0.05), and TEA episodes (*F* = 13.437, *p* < 0.01).

Oral administration of the methanolic extract of *S. hortensis* (200 mg/kg/d), when applied alone, despite the fact that it was not statistically different, decreased all estimated parameters in OF and EPM tests when compared to the control. However, SH, continuously applied before and following cisplatin application in different doses, induced the alteration in cisplatin-induced anxiogenic effect. The attenuation of cisplatin-induced anxiogenic effect in OF and EPM tests was observed only in E100 and was manifested by a significant anxiolytic-like effect when compared to the cisplatin group (*p* < 0.01), reversing CDCZ and FCZ (Figures 1(a) and 1(b)) in OF and CDOA and FOA (Figures 2(a) and 2(b)) in EPM to the control values. SH in the lowest (50 mg/kg/d) and the highest (200 mg/kg/d) applied doses was not sufficient to abolish the anxiogenic effect of cisplatin by means of the principal indicators of anxiety in both tests. The similar consequences of SH were manifested on locomotor activity in both tests. The orally applied extract in a dose of 100 mg/kg/d diminished cisplatin-induced decline in TDM and %TM in both OF (Figures 1(c) and 1(d)) and EPM (Figures 2(c) and 2(d)) tests. SH in this dose applied with cisplatin significantly increased TDM in both tests (Figures 1(c) and 1(d) and Figures 2(c) and 2(d)) compared to the lower and higher doses (*p* < 0.01), while this effect was not significant for %TM with the extract in a higher dose. However, both the locomotor activities in both tests for the animals treated with the higher and lower doses of the extract remained significantly diminished compared to the control values (*p* < 0.01) except for %TM in EPM for the higher dose. SH, when applied with cisplatin in the dose of 100 mg/kg/d, was sufficient to attenuate the cisplatin-induced decline in exploratory activity in both OF and EPM tests (Figure 1(e) and Figures 2(e)–2(g)). The lower dose of extract (50 mg/kg/d) failed to ameliorate this manifestation of anxiety-like behavior, which was manifested by significantly lower exploratory activity compared to the control values (*p* < 0.01), and even when compared to the effect of the middle dose of the extract (*p* < 0.01 for OF and TEA in EPM). Although the exploratory activity following the administration of the higher dose (200 mg/kg/d) along with cisplatin was not significantly lower compared to the middle dose (100 mg/kg/d), except for TEA, this anxiogenic feature of simultaneous administration of cisplatin and SH extract in the dose of 200 mg/kg/d remained significantly below the control values (*p* < 0.01), except for the number of rearings in EPM (Figure 2(f)).

When compared to SH, silymarin (a standardized plant extract with proven antioxidant effects *in vivo*) administration in a dose of 100 mg/kg/d showed similar protective effect on behavioral alterations induced by cisplatin in OF and EPM to the equivalent dose of SH (Figures 1 and 2) but still more pronounced than the effects of lower (50 mg/kg/d) and higher (200 mg/kg/d) doses of extract. As shown in Figure 2(a), silymarin did not prevent a decline in CDOA (*p* < 0.01) after cisplatin administration when compared to the control and did not produce a significant increase in any of the principle parameters for anxiety estimation in the applied tests. The protective effect following cisplatin application of silymarin was more pronounced by means of locomotor activity estimation, where it significantly increased cisplatin-induced decline in TDM (Figure 1(c) and Figure 2(c), *p* < 0.01), as well as exploratory activity in both tests (Figure 1(e) and Figures 2(e)–2(g), *p* < 0.01), except for the number of rearings in EPM.

### 3.2. *Satureja hortensis* L. Extract Prevents Oxidative Damage Induced by Cisplatin

**Figure 3:**
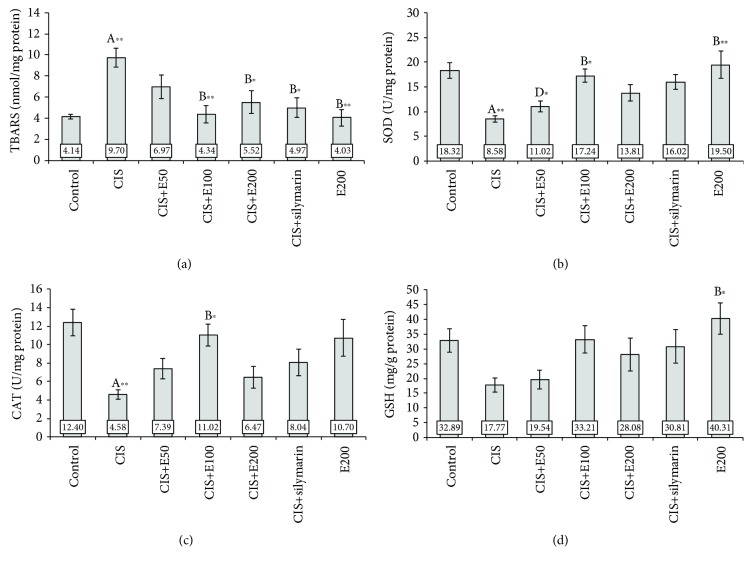
Oxidative stress markers in the rat hippocampus: (a) TBARS, (b) SOD, (c) CAT, and (d) GSH. Values are the mean ± standard error of the mean (SEM); *n* = 5 per group. ∗ denotes a significant difference of *p* < 0.05; ∗∗ denotes a significant difference of *p* < 0.01. A: control vs. other groups; B: CIS vs. other groups; C: CIS+silymarin vs. other groups; D: E200 vs. other groups; E: CIS+E50 vs. CIS+E100; F: CIS+E50 vs. CIS+E200; G: CIS+E100 vs. CIS+E200.

All applied protocols resulted in significant alterations in oxidative stress markers: TBARS (*F* = 5.400, df = 6), SOD (*F* = 6.103), and CAT (*F* = 4.396). A single application of cisplatin (7.5 mg/kg b.w.) caused a significant (*p* < 0.01) increase in TBARS and decline in SOD and CAT activity (*p* < 0.01) in the hippocampus, when compared to the control group (Figures 3(a)–3(c), respectively). Except for the lowest estimated dose of *S. hortensis* extract (50 mg/kg b.w.), all tested concentrations of extract and silymarin provided a significant amelioration in the level of TBARS, in comparison with the cisplatin group. The application of SH at a dose of 100 mg/kg b.w. showed the most pronounced (*p* < 0.01) effect on the decrease of the TBARS level in hippocampal tissue in comparison with the cisplatin group. At the same time, the increase of SOD activity in the hippocampus, when compared to the cisplatin group, following administration of SH extracts was significant (*p* < 0.05) only with the dose of 100 mg/kg (Figure 3(b)). The activity of CAT in hippocampal tissue (Figure 3(c)) also significantly increased (*p* < 0.05) only in the extract-treated group at a dose of 100 mg/kg b.w., when compared to the cisplatin group. On the other hand, the GSH level in the hippocampus was decreased, although not significantly, by a single administration of cisplatin. However, the data presented in Figure 3(d) showed improvements in the GSH level in the groups treated with higher doses of SH (100 and 200 mg/kg) as well as with silymarin, but these effects were not significantly pronounced (*F* = 3.034). The level of GSH in the extract per se group was slightly increased (*p* > 0.05), compared to the control, and as such is statistically different from the cisplatin group (*p* < 0.05).

The application of silymarin (100 mg/kg b.w.) in combination with cisplatin showed almost equal effects in the regulation of SOD and CAT activities in the hippocampus compared to *S. hortensis* extract at a dose of 100 mg/kg b.w. Application of extract at a dose of 200 mg/kg b.w. in combination with cisplatin was less effective in the regulation of all examined oxidative stress-related parameters compared to the extract dose of 100 mg/kg b.w. No significant changes were observed among the examined antioxidant hippocampal tissue parameters between the control group and the group treated with extract at a dose of 200 mg/kg b.w. without cisplatin application.

### 3.3. Diminishing of Cisplatin-Induced Proapoptotic Actions by Antioxidant Supplementation

**Figure 4:**
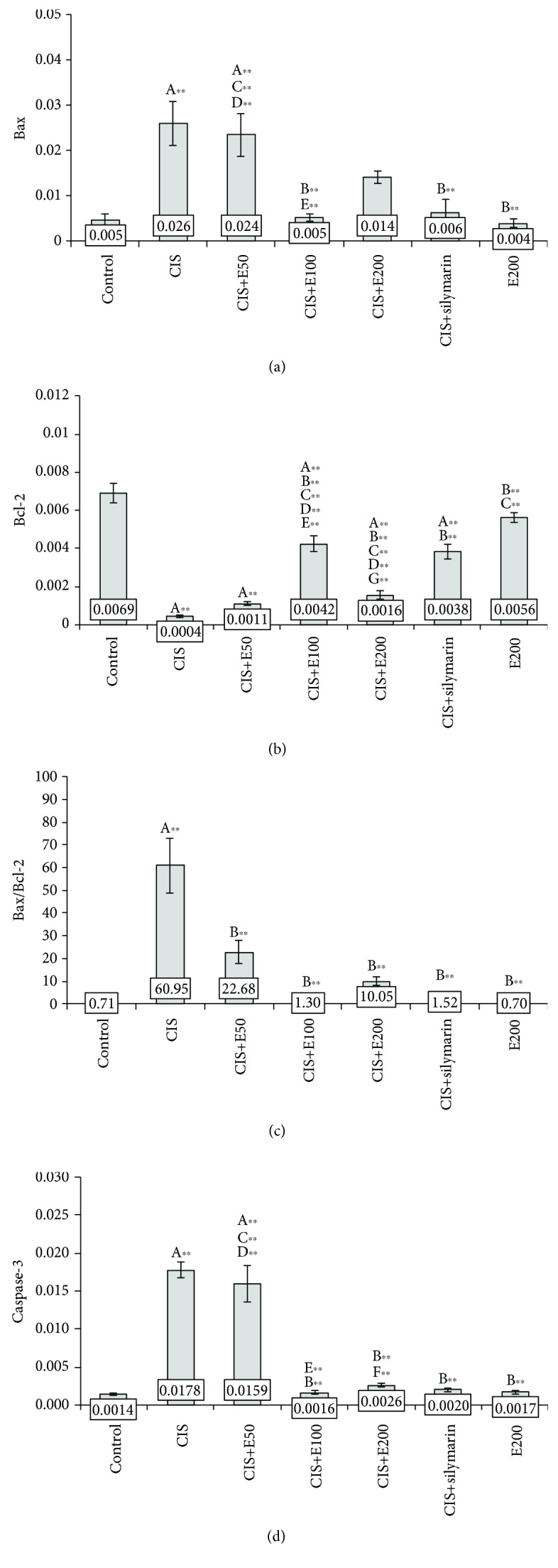
The relative expression of genes related to apoptosis in the rat hippocampus: (a) Bax, (b) Bcl-2, (c) Bax/Bcl-2 ratio, and (d) caspase-3. Values are the mean ± standard error of the mean (SEM); *n* = 5 per group. ∗ denotes a significant difference of *p* < 0.05; ∗∗ denotes a significant difference of *p* < 0.01. A: control vs. other groups; B: CIS vs. other groups; C: CIS+silymarin vs. other groups; D: E200 vs. other groups; E: CIS+E50 vs. CIS+E100; F: CIS+E50 vs. CIS+E200; G: CIS+E100 vs. CIS+E200.

The protocols applied in this study significantly altered the relative expression of genes involved in the regulation of cellular apoptosis, Bax (*F* = 10.165, df = 6), and Bcl-2 (*F* = 58.950) in hippocampal tissue. As shown in Figure 4, the application of cisplatin in the single dose induced a significant increase in Bax and decline in Bcl-2 relative gene expression compared to the control (*p* < 0.01), which resulted in significant augmentation of the Bax/Bcl-2 ratio (*F* = 19.754, *p* < 0.01). Although oral administration of SH did not significantly affect the hippocampal expression of those genes when applied alone, simultaneous oral intake of *S. hortensis* extract in a dose of 100 mg/kg reduced Bax and enhanced Bcl-2 hippocampal gene expression (*p* < 0.01), reversing the cisplatin-induced effects to control values (Figures 4(a) and 4(b)). On the other hand, administration of both lower and higher doses of *S. hortensis* extract (50 and 200 mg/kg) was not sufficient to significantly alter individual gene expression of Bax and Bcl-2 (Figure 4) but still significantly declined the Bax/Bcl-2 ratio in hippocampal tissue (*p* < 0.01). The effects of SH in a dose of 100 mg/kg/d on cisplatin-induced alterations of Bax and Bcl-2, as well as their ratio (*p* < 0.01), compared to the CIS group were very similar to the effects of simultaneous administration of silymarin in an equal dose. The relative gene expression of caspase-3, an apoptosis coordination enzyme, was also significantly affected by the applied protocols (*F* = 53.467) in the same manner as Bax. Thus, cisplatin administration significantly increased caspase-3 hippocampal expression (Figure 4(d)) compared to the control (*p* < 0.01). This manifestation of cisplatin proapoptotic action was lowered with all orally administered extracts (CIS+E100, CIS+E200, and CIS+silymarin) compared to the CIS group (*p* < 0.01), except for the lowest dose of *S. hortensis* extract (50 mg/kg/d).

**Figure 5:**
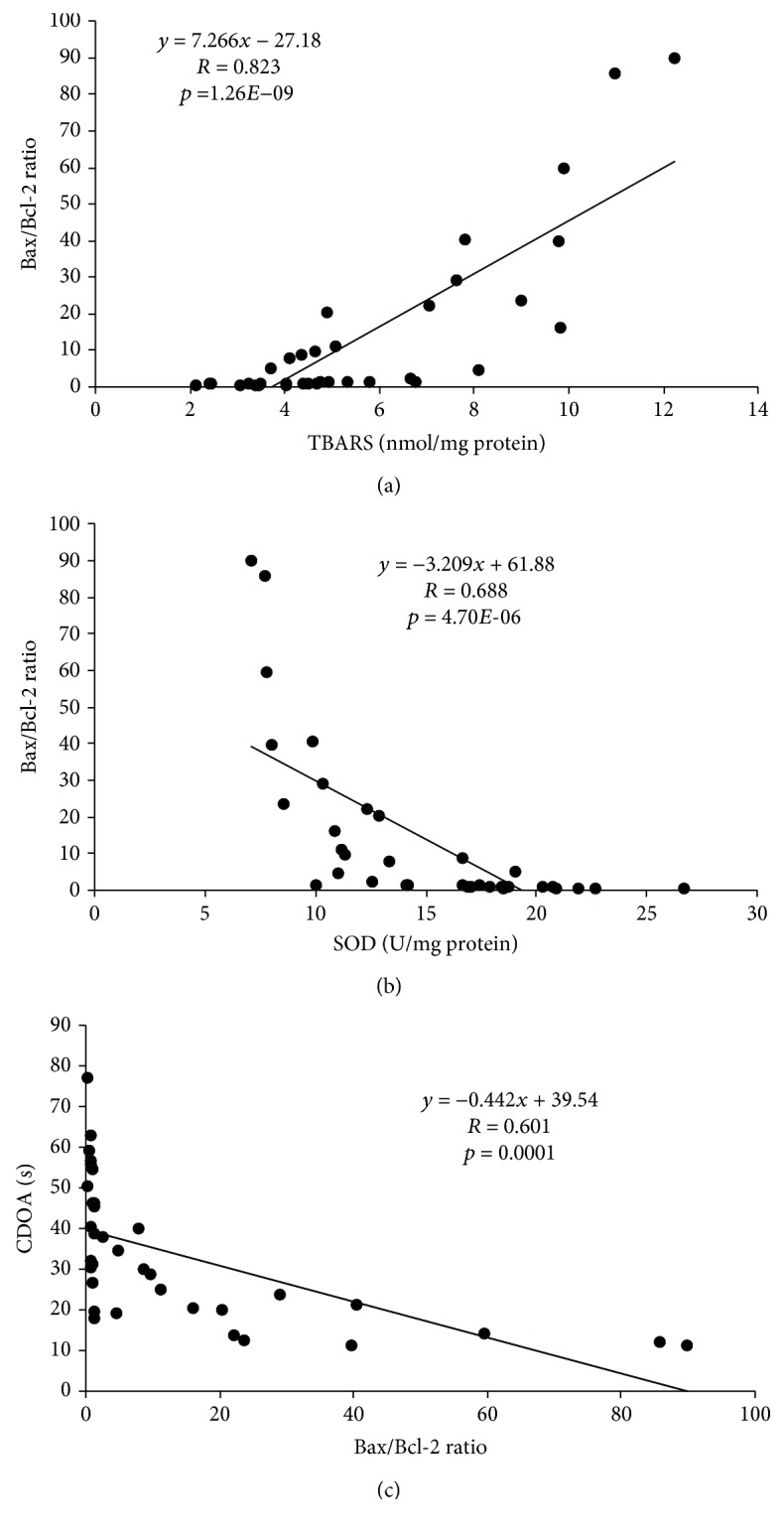
The relationship between (a) TBARS and Bax/Bcl-2 ratio, (b) SOD activity and Bax/Bcl-2 ratio, and (c) Bax/Bcl-2 ratio and CDOA in hippocampal tissue (*n* = 35).

As shown in Figure 5(a), simple regression analysis revealed a strong positive correlation between the index of lipid peroxidation expressed as TBARS and the Bax/Bcl-2 ratio (*R* = 0.823, *p* = 1.26*E*‐09). The analysis also indicated strong but negative (Figure 5(b)) correlation between the hippocampal antioxidant capacity estimated by means of SOD activity and Bax/Bcl-2 ratio (*R* = 0.688, *p* = 4.70*E*‐06). Finally, the analysis presented in Figure 5(c) confirms a strong, also negative, correlation between the Bax/Bcl-2 ratio and the principal indicator of the increased anxiety level obtained in the EPM test expressed as CDOA (*R* = 0.601, *p* = 0.0001).

## 4. Discussion

Although chemotherapy is one of the most employed approaches in the treatment of numerous malignancies, this therapeutic approach usually results in numerous adverse effects. Cisplatin, a widely used chemotherapeutic drug, has also been reported for serious clinical side effects, including severe manifestations of neurotoxicity, despite the fact that it poorly crosses the blood-brain barrier under physiological conditions [27], such as psychological, cognitive, and perceptual impairment [6, 28, 29]. The manifestations of neurotoxicity following cisplatin administration have also been confirmed in animal experimental models. Cisplatin-induced behavioral alterations include cognitive dysfunction [30] and depressive- and anxiety-like behaviors in rats [31, 32]. The results obtained in this study showed a clear anxiogenic effect of cisplatin (Figures 1 and 2). The anxiety-like behavior following cisplatin administration was manifested by means of the decline of the most convincing indicators in OF (CDCZ and FCZ) and EPM (CDOA and FOA) tests and confirmed by a decrease in locomotor and exploratory activities, in both tests, which is typical for anxiogenic features [33]. The anxiogenic effect of cisplatin applied in the single dose (7.5 mg/kg b.w.), as observed in this study, is in accordance with previously reported anxiety-like behavior following chronic cisplatin administration (5 mg/kg for five weeks and seven weeks) [5, 7].

Behavioral manifestations of cisplatin-induced neurotoxicity occur simultaneously with alterations in the hippocampus that involve inhibition in cell proliferation, differentiation, and neurogenesis with alterations in neurotransmitter content with morphological verification of neuronal damage [34–36]. Cisplatin also causes apoptosis [37], oxidative stress [38], and inflammation [39]. Our results show that the anxiogenic effect of cisplatin was accompanied by significant alterations in hippocampal tissue oxidative status (Figure 3). The increased lipid peroxidation following cisplatin administration observed in this study, as well as the decline in the antioxidant defense system, expressed by means of SOD and CAT activity in the hippocampus, is in line with reported alterations in oxidative status following chronic cisplatin treatment [7]. However, the lack of significant alteration in total hippocampal glutathione after a single dose of cisplatin, which is not in accordance with the reduction in GSH reported in that study, may be explained by different experimental protocols that included prolonged cisplatin treatment (seven weeks), sufficient to induce a significant decline in hippocampal glutathione. The postulated mechanisms of cisplatin neurotoxic effects in the rat hippocampus include the action via alteration in the expression of genes involved in antioxidative defense responses [7]. Changes in gene expression provoked by cisplatin implicate both downregulation of Nrf2, as a key regulator of protection against oxidative stress [40], and HO-1 (controlled by the Nrf2 gene level), as well as upregulation of NF-*κ*B, which is responsible for cell damage in a various manner, including the increase of proinflammatory cytokines. Those mechanisms may lead to neuroinflammatory cascade in the hippocampus with the consequent behavioral deficits [41]. Inflammatory mediators have been reported to reduce hippocampal BDNF levels [42], which may result in a decrease of hippocampal volume that, in turn, may be the principal cause of various mood disorders [43], including anxiety-like behavior.

Not surprisingly for the anticancer drug, cisplatin administration in this study exerted proapoptotic response (Figure 4). This action was manifested by both an increase in Bax and caspase-3 relative gene expression and a decline in Bcl-2 hippocampal expression. The significant alterations in apoptotic gene relative expression in the hippocampus were confirmed by a typical proapoptotic shift in the gene expression ratio, as the intracellular ratio of Bax and Bcl-2 stems from their antagonistic action in apoptosis control and describes the ability of a cell to respond to an apoptotic signal [44, 45]. A similar response to single cisplatin application was reported by Manohar and coworkers [8]. That comprehensive study offered excellent insight in the individual proapoptotic gene expression pattern in a rat hippocampus, showing that the whole proapoptotic set of genes (Bid, Bik, and Bok) was elevated following cisplatin, while the antiapoptotic capacity of hippocampal neurons, expressed as Bcl2a1 relative gene expression, was simultaneously significantly deprived along with the marker of cell proliferation (Ki67). The proapoptotic outcome provoked by cisplatin, as observed in our study, accompanied by the previously described oxidative damage in the hippocampus, may be considered as potential biological mechanisms involved in emotional impairment manifested by anxiety-like behavior [5, 6].

Previously, we demonstrated that *S. hortensis* methanol extract used in this study was rich in phenolic compounds, particularly in rosmarinic acid (24.9 mg/g of dry extract) [17]. Several phenolic acids, such as chlorogenic, caffeic, syringic, *p*-coumaric, sinapic, and isoferulic acids, were identified in lower concentrations, as well as a certain amount of flavonoids (quercetin, apigenin, luteolin, and naringenin) and their derivatives [17]. Rosmarinic acid is mostly known for its antioxidant features [46], but it has shown many biological activities including cytotoxicity, antimicrobial, antiviral, antidiabetic, anti-inflammatory, antiallergic, and immunomodulatory activities [47–50]. In previous investigations, rosmarinic acid showed significant alleviation of oxidative stress and anti-inflammatory potential *in vivo* [47]. Especially important were antioxidant properties of rosmarinic acid during *in vivo* cisplatin treatment [48, 51]. Domitrović and coworkers proposed that protective effects of rosmarinic acid against CP-induced nephrotoxicity included reducing the levels of oxidative stress markers where rosmarinic acid decreased lipid peroxidation, 4-HNE, CYP2E1, and HO-1 immunoreactivity [48]. Also, this phenolic acid had an influence on inflammatory response in kidneys by lowering the expression of NF-*κ*B p65 and TNF-*α* and reducing the parameters of apoptotic cell death (expression of p53 and p-p53) compared to the cisplatin treatment. A study of Lee and coworkers showed that rosmarinic acid was able to protect human dopaminergic neuronal cells against H_2_O_2_-induced oxidative stress by regulating the apoptotic process and suggested that this compound may be used in the prevention of neurodegenerative diseases [52]. Also, rosmarinic acid showed neuroprotective properties against H_2_O_2_-induced oxidative damage in astrocytes [53].

The restoration of equilibrium in the level of intrinsic antioxidant defense armory in tissue in cisplatin-treated rats after oral treatment with summer savory extract has been proposed as a dominant ameliorating mechanism [17]. The extract of *S. hortensis* in this study was able, not surprisingly, to reduce oxidative stress and apoptotic gene relative expression in the hippocampal tissue of experimental animals. Therefore, the neuroprotective effects of summer savory may be related to the presence of a high amount of rosmarinic acid in the extract [17]. Moreover, other phenolic compounds detected in *S. hortensis* showed significant *in vivo* antioxidant potential during the treatment with cisplatin. For example, quercetin [54] and naringenin [55] reduced cisplatin-induced nephrotoxicity while luteolin inhibited the accumulation of platinum, inflammation, and apoptosis in renal tissues during treatment with cisplatin [56].

Accompanying oxidative stress, mitochondrial damage and induced cell death may be involved in neurological disorders and neural dysfunction [57]. In our study, a single application of cisplatin increased the expression of proapoptotic Bax gene in the hippocampus, in response to the increased level of ROS. Additionally, cisplatin downregulated the antiapoptotic Bcl-2 gene, which is in line with previous studies [8]. Oral administration of *S. hortensis* extract (100 mg/kg/d), as well as silymarin, significantly changed the balance between Bcl-2 and Bax expression, indicating that the apoptosis in the hippocampus was inhibited by the application of the extract. Our results were in accordance with another research, which demonstrated that *S. hortensis* extract controlled apoptosis in H_2_O_2_-challenged Jurkat cells [18]. Considering the positive correlation observed between the Bax/Bcl-2 ratio and TBARS level, it could be concluded that the main denominator of the apoptotic pathway may be lipid peroxidation, which is the main factor in disturbance of cellular integrity.

The plants from genus *Satureja* (Lamiaceae) are known for their various pharmacological activities, including antioxidant, anti-inflammatory, immunostimulant, hepatoprotective, and anticancer properties [16, 58], but the studies regarding the behavioral effects of *Satureja* species have not been reported so far. In this study, for the first time, it was shown that the *Satureja* species may also have an influence on the reduction of cisplatin-induced neurotoxicity. The effects of *S. hortensis* extract on the regulation of oxidative stress and proapoptotic factors in the hippocampal tissue were comparable to silymarin. When applied in the same doses, both exhibited very similar effects on these parameters. Silymarin, as a standardized herbal preparation known for its antioxidant effects, was able to reduce the toxic effects caused by many xenobiotics, including drug-induced toxicity [59]. The protective effects of silymarin on cisplatin-induced ototoxicity [60], as well as liver, kidney, and testicular toxicities, have been reported so far [17].

In summary, anxiogenic action of cisplatin, accompanied with increased oxidative stress and proapoptotic manifestations in the hippocampus, can be attenuated by antioxidant supplementation with a simultaneous diminishing of oxidative damage and enhanced antiapoptotic capacity in rats, suggesting a potential beneficial role of antioxidant-rich natural products in the treatment of cisplatin-induced neurotoxicity patterns.

## Data Availability

The data used to support the findings of this study are available from the corresponding author upon request.

## Supplementary Materials

Supplementary Materials Supplement 1: RT-PCR primers used in this study.
