# Supplementary material for: Antioxidant Effects of Satureja hortensis L. Attenuate the Anxiogenic Effect of Cisplatin in Rats
Source: Oxid Med Cell Longev. 2019 Jul 29;2019:8307196. doi: 10.1155/2019/8307196 (PMC6701305; doi:10.1155/2019/8307196)
Supplement: Supplementary Materials — Supplement 1: RT-PCR primers used in this study. [file 8307196.f1.docx]

| Name |  | Sequence (5' to 3') |
| --- | --- | --- |
| β-actin | F | GATCAGCAAGCAGGAGTACGAT |
|  | R | GTAACAGTCCGCCTAGAAGCAT |
| Bax | F | GCTACAGGGTTTCATCCAGGAT |
|  | R | ATGTTGTTGTCCAGTTCATCGC |
| Bcl-2 | F | GCAAAGCACATCCAATAAAAGCG |
|  | R | GTACTTCATCACGATCTCCCGG |
| Caspase-3 | F | GGAAGATCACAGCAAAAGGAGC |
|  | R | GCAGTAGTCGCCTCTGAAGAAA |

**Supplement 1**
